# Nasal Polypous

**Published:** 1867-09

**Authors:** R. P. Hunt

**Affiliations:** Chicago


### NASAL POLYPUS.

By R. P. Hunt, M.D., of Chicago.

Dear Doctor:—

When the late civil war, or rebellion, closed, I was on duty in hospital, at Columbus, Ga. The surrender having taken place, I, like all others, concluded to reach my own loved ones, whom for years I had not seen. Accordingly, I left Columbus, where I was stationed, and wended my way towards Louisville, where my family was. In returning, it was necessary to repass ground once before passed.

On reaching Griffin, Ga., where I had been previously stationed, I found surgeons of long-standing in the rebel army, like myself, thrown out. It was necessary to remain there, but whilst there, a singular case occurred:—

I was called in consultation by one of the Confederate surgeons, upon a case of most peculiar character. What was it? A ease of nasal polypus.

Some months previously the soldier presented himself at the hospital for treatment; it was then found impossible to remove the polypus as it is ordinarily done. The nose was necessarily split on the side requiring operation, and the excrescence removed. The tumor returned.

A few months after, when there was no Dixie, and I was wending my weary way towards my family and home, if I had a home, I was compelled to stop a few days at Griffin, where I previously had been on duty. Whilst there, a fellow-surgeon called my attention to this case.

The war was over, and all hospital accommodation broken up; yet it did not enter the mind of a medical gentleman to see suffering and not attempt to relieve it. Being there, the medical officer who had previously treated the case requested me to examine it. Thus it was simply a case of charity, and not of army surgery.

After a close and minute examination, I found out that the patient could not sleep in a recumbent position. He was compelled, by the pressure upon his epiglottis, to remain in a chair, as are many cases of asthma. A slight doze, and he would fall and bruise himself. Sleep was really no rest to him; he was young and otherwise vigorous. The case was urgent. Having no means of treating him, we advised that he should pass on to Nashville, enter the hospital, and be well and properly treated. No! he had no money, and could not walk that distance. Like him, we had no money; so what was to be done? The question was ultimately referred to me for a final decision. And now for the case, as I think I have wasted enough of my time and your pages.

The boy came in; I examined him, and saw from the effects of the previous operation that it had been totally ineffectual. My opinion was asked. It is needless to say, the war being over, there being no hospital accommodations, no means of caring for the sick, I agreed with those who wished to send him to a Nashville hospital for treatment. To this he objected strenuously, giving as reasons his utter disbelief in the possibility of reaching there, that he could not lie down and sleep, that he was suffocating, and that unless relieved he would prefer death immediately. Called upon for my opinion, I first agreed with those preceding me, it was best, if possible, to reach a hospital. To this advice he would not listen. Then, what to do?

The left nasal passage was entirely filled, neither forceps nor probe could pass. The polypus was hard and unyielding; the patient was almost suffocating. He claimed relief, and claimed it as a right. There was but one means to resort to; the previous operation had been fruitless in its results, a new one was necessary. What was it to be?

On an examination, as careful as I could make, I suggested the possibility of the polypus having forced its way into the highmorianum antrum. Thence, the operation to be performed: first, slit up the nose, remove the nasal portion of the polypus, and verify or falsify the diagnosis; if correct, the superior maxillary must be removed. Well, the patient was informed of all his chances, and still advised to proceed to Nashville and enter a hospital. No! "I will die before reaching there, and prefer to die in making an effort to live; cut on." Finding this courage, this indomitable will, the operation was performed. The whole of the left nares was occupied by a substance fully as large as the egg of a pullet, as a matter of course a pullet's egg somewhat elongated; it was thoroughly impacted, reaching from the external orifice back to the pharynx; it also, as had been foreseen, branched off into the antrum. On removing the superior maxillary, the cavity was found filled to its utmost capacity. The excrescence was of a scirrhus character, evidently malignant. The result was what he wished—death.

Prof. Gross says, "there are only two" special forms, "the gelatinous and fibrous." "The *fibrous polypus* occurs at nearly every period of life. * * * * For the removal of the *naso-pharyngeal* polypus two distinct operations have been proposed: one by Nélaton, * * * the other by Flambert, of Lyons, consisting in the excision of the upper jaw, first practiced by him in 1840, and now recognized as a perfectly legitimate procedure."

This information was not before us at the time the case i question plead for relief.

